# Use of Lactic Acid Bacteria to Reduce Methane Production in Ruminants, a Critical Review

**DOI:** 10.3389/fmicb.2019.02207

**Published:** 2019-10-01

**Authors:** Natasha Doyle, Philiswa Mbandlwa, William J. Kelly, Graeme Attwood, Yang Li, R. Paul Ross, Catherine Stanton, Sinead Leahy

**Affiliations:** ^1^Teagasc Moorepark Food Research Centre, Fermoy, Ireland; ^2^School of Microbiology, University College Cork, Cork, Ireland; ^3^Donvis Ltd., Palmerston North, New Zealand; ^4^AgResearch Limited, Grasslands Research Centre, Palmerston North, New Zealand; ^5^APC Microbiome Ireland, University College Cork, Cork, Ireland

**Keywords:** lactic acid bacteria, methane, methanogens, bacteriocins, direct-fed microbials, silage inoculants, mitigation

## Abstract

Enteric fermentation in ruminants is the single largest anthropogenic source of agricultural methane and has a significant role in global warming. Consequently, innovative solutions to reduce methane emissions from livestock farming are required to ensure future sustainable food production. One possible approach is the use of lactic acid bacteria (LAB), Gram positive bacteria that produce lactic acid as a major end product of carbohydrate fermentation. LAB are natural inhabitants of the intestinal tract of mammals and are among the most important groups of microorganisms used in food fermentations. LAB can be readily isolated from ruminant animals and are currently used on-farm as direct-fed microbials (DFMs) and as silage inoculants. While it has been proposed that LAB can be used to reduce methane production in ruminant livestock, so far research has been limited, and convincing animal data to support the concept are lacking. This review has critically evaluated the current literature and provided a comprehensive analysis and summary of the potential use and mechanisms of LAB as a methane mitigation strategy. It is clear that although there are some promising results, more research is needed to identify whether the use of LAB can be an effective methane mitigation option for ruminant livestock.

## Introduction

While ruminant animals play an important role in sustainable agricultural systems (Eisler et al., 2014) they are also an important source of greenhouse gas (GHG) emissions (Reisinger and Clark, 2018). Regardless of the ruminant species, the largest source of GHG emissions from ruminant production is methane (CH_4_), with more than 90 percent of emissions originating from enteric fermentation (Opio et al., 2013). Enteric fermentation is a digestive process by which a community of microbes present in the forestomach of ruminants (the reticulo-rumen) break down plant material into nutrients that can be used by the animal for the production of high-value proteins that include milk, meat and leather products. Hydrogen (H_2_) and methyl-containing compounds generated as fermentation end products of this process are used by different groups of rumen methanogenic archaea to form CH_4_, which is belched and exhaled from the lungs via respiration from the animal and released to the atmosphere. In the coming decades, livestock farmers will face numerous challenges and the development of technologies and practices which support efficient sustainable food production while moderating greenhouse gas emissions are urgently required. More than 100 countries have committed to reducing agricultural GHG emissions in the 2015 Paris Agreement of the United Nations Framework Convention on Climate Change, however, known agricultural practices could deliver just 21–40% of the needed reduction, even if implemented fully at scale (Wollenberg et al., 2016). New technical mitigation options are needed. Reviews of CH_4_ mitigation strategies consistently discuss the possibility that lactic acid bacteria (LAB) could be used to modulate rumen microbial communities thus providing a practical and effective on-farm approach to reducing CH_4_ emissions from ruminant livestock (Hristov et al., 2013; Takahashi, 2013; Knapp et al., 2014; Jeyanathan et al., 2014; Varnava et al., 2017). This review examines the possible contribution of LAB in the development of an on-farm CH_4_ mitigating strategy.

## Results and Discussion

### General Characteristics of Lactic Acid Bacteria

Lactic acid bacteria are Gram positive, acid tolerant, facultatively anaerobic bacteria that produce lactic acid as a major end-product of carbohydrate fermentation (Stilez and Holzapfel, 1997). Biochemically they include homofermenters that produce primarily lactic acid, and heterofermenters that also give a variety of other fermentation end-products such as acetic acid, ethanol and CO_2_. LAB have long been used as starter cultures for a wide range of dairy, meat and plant fermentations, and this history of use in human and animal foods has resulted in most LAB having Qualified Presumption of Safety (QPS) status in the European Union or Generally Recognized as Safe (GRAS) status in the United States. The main LAB genera used as starter cultures are *Lactobacillus*, *Lactococcus*, *Leuconostoc*, and *Pediococcus* (Bintsis, 2018) together with some species of *Enterococcus* and *Streptococcus*.

In addition to their contribution to the development of food flavor and texture, LAB have an important role in inhibiting the growth of spoilage organisms through the production of inhibitory compounds. These compounds include fermentation products such as organic acids and hydrogen peroxide as well as ribosomally synthesized peptides known as bacteriocins (Cotter et al., 2013). In many cases, the physiological role of bacteriocins is unclear but they are thought to offer the producing organism a competitive advantage, via their ability to inhibit the growth of other microorganisms, particularly in complex microbial communities. Some strains also produce other compounds such as non-ribosomally synthesized peptides which may have additional antimicrobial activity (Mangoni and Shai, 2011).

In recent years much interest has been shown in the use of LAB as probiotic organisms and in their potential contribution to human health and well-being. LAB have also been advocated as probiotics to improve food animal production and as alternatives to antibiotics used as growth promotors (Vieco-Saiz et al., 2019).

### LAB and the Rumen

LAB are members of the normal gastrointestinal tract microbiota, however, in ruminants these organisms are generally only prevalent in young animals before the rumen has properly developed (Stewart et al., 1988). LAB are unable to initiate the metabolism of plant structural polysaccharides and are not regarded as major contributors to rumen fermentation. In the Global Rumen Census project (Henderson et al., 2015) which profiled the microbial community of 684 rumen samples collected from a range of ruminant species, only members of the genus *Streptococcus* were found in a majority of samples (63% prevalence, 0.5% abundance). Nevertheless, LAB can be readily isolated from the rumen, with some species such as *Lactobacillus ruminis* and *Streptococcus equinus* (formerly *S. bovis*) being regarded as true rumen inhabitants while others (*Lactobacillus plantarum* and *Lactococcus lactis*) are likely to be transient bacteria that have been introduced with the feed (Stewart, 1992). Several obligately anaerobic rumen bacteria also produce lactate as a fermentation end product and two of these are included in this review. These organisms (*Kandleria vitulina* and *Sharpea azabuensis*) are both members of the family *Erysipelotrichaceae* within the phylum Firmicutes, although *Kandleria vitulina* was formerly known as *Lactobacillus vitulinus* (Salvetti et al., 2011). *Sharpea* and *Kandleria* are a significant component of the rumen microbiome in low CH_4_ yield animals in which rapid heterofermentative growth results in lactate production (Kamke et al., 2016).

Table 1 lists the rumen LAB together with strains of *Kandleria* and *Sharpea* that have been genome sequenced along with potential antimicrobial biosynthetic clusters predicted from the genome sequence data. The majority (81%) of genome sequenced strains from rumen members of the *Streptococcaceae* encode antimicrobial biosynthetic clusters, and previous studies have also reported that rumen streptococci can produce a range of bacteriocins (Iverson and Mills, 1976; Mantovani et al., 2001; Whitford et al., 2001). Conversely, antimicrobial biosynthetic genes have not been identified from the species *Kandleria vitulina* and *Sharpea azabuensis*.

**TABLE 1:** List of rumen LAB cultures in addition to a further two species of obligately anaerobic rumen bacteria (*Kandleria* and *Sharpea*) also known to produce lactate as a fermentation end product.

**Family/Order**	**Genus/Species**	**Strain**	**Culture collection #**	**Origin**	**Comments**	**Predicted antimicrobial biosynthetic clusters**	**References**
Enterococcaceae	*Enterococcus faecalis*	68A		Sheep rumen/NZ			Hudson et al., 1995
Enterococcaceae	*Enterococcus gallinarum*	SKF1		Sheep rumen/NZ		Lantipeptide	Morvan and Joblin, 2000
Enterococcaceae	*Enterococcus mundtii*	C2		Cow rumen/NZ		Bacteriocin	
Enterococcaceae	*Enterococcus* sp.	KPPR-6		Cow rumen/NZ		Bacteriocin, NRPS	
Erysipelotrichaceae	*Kandleria vitulina*	MC3001		Cow rumen/NZ			Noel, 2013
Erysipelotrichaceae	*Kandleria vitulina*	WCE2011		Cow rumen/NZ			Noel, 2013
Erysipelotrichaceae	*Kandleria vitulina*	RL2	DSM 20405	Calf rumen/UK	Type strain		Bryant et al., 1958; Sharpe et al., 1973
Erysipelotrichaceae	*Kandleria vitulina*	S3b		Sheep rumen/NZ			Attwood et al., 1998
Erysipelotrichaceae	*Kandleria vitulina*	WCC7		Cow rumen/NZ			
Erysipelotrichaceae	*Kandleria vitulina*	KH4T7		Cow rumen/NZ			
Erysipelotrichaceae	*Sharpea azabuensis*	RL1	DSM 20406	Calf rumen/USA			Bryant et al., 1958
Erysipelotrichaceae	*Sharpea azabuensis*	KH1P5		Cow rumen/NZ			
Erysipelotrichaceae	*Sharpea azabuensis*	KH2P10		Cow rumen/NZ			
Lactobacillaceae	*Lactobacillus brevis*	AG48		Sheep rumen/NZ		Lantipeptide	Hudson et al., 2000
Lactobacillaceae	*Lactobacillus mucosae*	AGR63		Cow rumen/NZ			Morvan and Joblin, 2000
Lactobacillaceae	*Lactobacillus mucosae*	WCC8		Cow rumen/NZ			
Lactobacillaceae	*Lactobacillus mucosae*	KHPC15		Cow rumen/NZ			
Lactobacillaceae	*Lactobacillus mucosae*	KHPX11		Cow rumen/NZ			
Lactobacillaceae	*Lactobacillus plantarum*	AG30		Sheep rumen/NZ			Hudson et al., 2000
Lactobacillaceae	*Lactobacillus ruminis*	RF1	DSM 20403	Cow rumen/UK	Type strain	Bacteriocin	Sharpe et al., 1973
Lactobacillaceae	*Lactobacillus ruminis*	WC1T17		Cow rumen/NZ			
Lactobacillaceae	*Lactobacillus ruminis*	RF3	ATCC 27782	Cow rumen/UK		Bacteriocin	Forde et al., 2011
Lactobacillaceae	*Pediococcus acidilactici*	AGR20		Sheep rumen/NZ			Morvan and Joblin, 2000
Streptococcaceae	*Lactococcus lactis* subsp. *cremoris*	DPC6856		Cow rumen/Ireland		Bacteriocin	Cavanagh et al., 2015
Streptococcaceae	*Lactococcus lactis* subsp. *lactis*	511		Cow rumen/NZ		Lantipeptide (nisin)	Kelly et al., 2010
Streptococcaceae	*Streptococcus equinus*	B315		Sheep rumen/NZ		Lantipeptide X2	Reilly et al., 2002
Streptococcaceae	*Streptococcus equinus*	SN033		Deer rumen/NZ		Lantipeptide X3	
Streptococcaceae	*Streptococcus equinus*	AG46		Sheep rumen/NZ			Hudson et al., 2000
Streptococcaceae	*Streptococcus equinus*	2B		Sheep rumen/UK			Oxford, 1958
Streptococcaceae	*Streptococcus equinus*	JB1		Cow rumen/USA		Bacteriocin	Russell and Baldwin, 1978
Streptococcaceae	*Streptococcus equinus*	GA-1		Cow rumen/NZ		Lantipeptide X2	
Streptococcaceae	*Streptococcus equinus*	pGA-7		Cow rumen/NZ		Bacteriocin, Lantipeptide	
Streptococcaceae	*Streptococcus equinus*	pR-5		Cow rumen/NZ		Lantipeptide	
Streptococcaceae	*Streptococcus equinus*	ES1		Sheep rumen/UK		Lantipeptide	Marounck and Wallace, 1984
Streptococcaceae	*Streptococcus equinus*	C277		Sheep rumen/UK		Bacteriocin, Lantipeptide	Wallace and Brammall, 1985
Streptococcaceae	*Streptococcus equinus*	H24		Calf rumen/USA		Lantipeptide	Boyer, 1969
Streptococcaceae	*Streptococcus equinus*	Sb04		Cow rumen/Australia		Bacteriocin	Klieve et al., 1999
Streptococcaceae	*Streptococcus equinus*	Sb05		Cow rumen/Australia		Bacteriocin	Klieve et al., 1999
Streptococcaceae	*Streptococcus equinus*	Sb10		Cow rumen/Australia		Bacteriocin, NRPS	Klieve et al., 1999
Streptococcaceae	*Streptococcus equinus*	Sb13		Cow rumen/Australia		Lantipeptide	Klieve et al., 1999
Streptococcaceae	*Streptococcus equinus*	Sb17		Cow rumen/Australia		Bacteriocin	Klieve et al., 1999
Streptococcaceae	*Streptococcus equinus*	Sb18		Cow rumen/Australia			Klieve et al., 1999
Streptococcaceae	*Streptococcus equinus*	Sb20		Cow rumen/Australia		Bacteriocin	Klieve et al., 1999
Streptococcaceae	*Streptococcus equinus*	YE01		Goat rumen/Australia			Klieve et al., 1999
Streptococcaceae	*Streptococcus equinus*	Sb09		Goat rumen/Australia		Bacteriocin	Klieve et al., 1999
Streptococcaceae	*Streptococcus equinus*	SI		Sheep rumen/Australia			Klieve et al., 1999
Streptococcaceae	*Streptococcus equinus*	AR3		Sheep rumen/Australia		Bacteriocin, Lantipeptide	Klieve et al., 1989
Streptococcaceae	*Streptococcus equinus*	HC5		Cow rumen/USA		Lantipeptide	Azevedo et al., 2015
Streptococcaceae	*Streptococcus gallolyticus*	TPC2.3	LMG 15572	Goat rumen/Australia		Bacteriocin	Brooker et al., 1994; Sly et al., 1997
Streptococcaceae	*Streptococcus henryi*	A-4		Cow rumen/NZ		Lantipeptide, Thiopeptide	

*All strains were sequenced as part of the Hungate1000 project (Seshadri et al., 2018) with the exceptions of *L. ruminis* RF3, *L. lactis* subsp. *cremoris* DPC68656 and *S. equinus* HC5.*

### How Are LAB Used in Ruminant Agriculture?

On-farm, LAB are used as direct-fed microbials (DFMs), probiotics and as silage inoculants. The terms DFM and probiotic are used interchangeably in animal nutrition and refer to any type of live microbe-based feed additive. Although the products have different purposes, there is considerable overlap in the bacterial species used.

The efficacy of DFMs containing LAB has been studied mostly in pre-ruminants where their reported benefits include a reduction in the incidence of diarrhea, a decrease in fecal shedding of coliforms, promotion of ruminal development, improved feed efficiency, increased body weight gain, and reduction in morbidity (Krehbiel et al., 2003). A meta-analysis of randomized controlled trials of LAB supplementation in young calves has shown that LAB can exert a protective effect and reduce the incidence of diarrhea (Signorini et al., 2012) and can increase body weight gain and improve feed efficiency (Frizzo et al., 2011). The meta-analysis further revealed that LAB can induce further beneficial effects if administered with whole milk and as a single strain inoculum. The use of DFM supplementation in young ruminants is expanding as farmers look to use natural alternatives to antibiotics to help improve calf health and promote growth.

In the adult ruminant, there is limited research available on the efficacy of LAB DFMs. Their use is targeted at improving the health and performance of animals (Table 2). With regard to health, a meta-analysis of trials evaluating the use of DFMs (predominantly *Lactobacillus*) to reduce the prevalence of *Escherichia coli* O157 fecal shedding in beef cattle has shown LAB supplementation to be efficacious (Wisener et al., 2015). Administration of *Lactococcus lactis* has been shown to be as effective as common antibiotics in the treatment of bovine mastitis (Klostermann et al., 2008). LAB DFMs have also been shown to minimize the risk of ruminal acidosis in some instances (Ghorbani et al., 2002; Lettat et al., 2012). A recent review by Rainard and Foucras (2018) appraised the use of probiotics for mastitis control. The authors concluded that based on the lack of scientific data the use of probiotics to prevent or treat mastitis is not currently recommended. However, use of teat apex probiotics deserves further research. The results from a small number of trials using only LAB supplementation treatment groups to enhance animal performance are mixed (Table 2). Studies where beneficial effects have been reported include an increase in milk yield, change in milk fat composition, improved feed efficiency, and increased daily weight gain but equally there have been studies where no change has been reported (see Table 2). Although responses to DFMs have been positive in some experiments, the basic mechanisms underlying these beneficial effects are not well defined or clearly understood.

**TABLE 2:** Animal trials which studied the effect of DFM supplementation containing LAB only on ruminant performance and health.

**Target**	**Genus**	**Sector**	**Animal**	**N**	**Treatment/Dose/Strain**	**Duration of trial**	**Effect**	**References Year**
Performance	*Lactobacillus plantarum Lactobacillus casei*	Dairy	Holstein cows	20	Treatments: (1) Control (2) 1.3 × 10^9^ cfu/g *Lactobacillus plantarum* P-8 *Lactobacillus casei* Zhang	30 days	LAB treatment increased milk produced and certain milk functional components (IgG, lactoferrin, lysozyme, lactoperoxidase)	Xu et al., 2017
Health	*Lactobacillus rhamnosus*, *Pedioccocus acidilactici*, *Lactobacillus reuteri*	Dairy	Holstein cows	20	Treatments given intravaginally: (1) *L. rhamnosus* CECT 278, *P. acidilactici* CECT 5915, and *L. reuter*i DSM 20016, with a final cell count of 4.5 × 10 ^10^cdu/dose and a relationship among the 3 probiotics of 12:12:1, respectively; (2) control.	3 weeks	Vaginal application of LAB maybe capable of modulating the pathogenic environment in the vaginal tract.	Genís et al., 2017
Performance	*Propionibacterium Lactobacillus plantarum Lactobacillus rhamnosus*	Dairy	Holstein cows	8	Treatments: (1) lactose (control); (2) 10^10^ cfu/d *Propionibacterium* P63; (3) 10^10^ cfu/d of both *Propionibacterium* P63 and *Lactobacillus plantarum* 115; (4) 10^10^ cfu/d of both *Propionibacterium* P63 and *Lactobacillus rhamnosus* 32	4 weeks	Some effects on CH_4_ production, ruminal PH and milk FA profile but results depended on DFM strain and diet.	Philippeau et al., 2017
Performance	*Lactobacillus acidophilus, Lactobacillus casei Bifidobacterium thermophilum Enterococcus*	Dairy	Ewes	16	Treatments: (1) control; (2) *Lactobacillus acidophilus* (2⋅5 × 10^7^ CFU/g), *Lactobacillus casei* (2⋅5 × 10^7^ CFU/g), *Bifidobacterium thermophilum* (2⋅5 × 10^7^ CFU/g), and *Enterococcus faecium* (2⋅5 × 10^7^ CFU/g	10 weeks	Supplementing ewes with DFM products has very minor effects on milk fatty acid profiles	Payandeh et al., 2017
Health	*Lactobacillus sakei Pediococcus acidilactici*	Dairy	Holstein cows	100	Treatments given intravaginally: (1 and 2) *L. sakei* FUA3089, *P. acidilactici* FUA3138, and *P. acidilactici* FUA3140 with a cell count of 10^8^ −10^9^ cfu/dose; (3) control	10 weeks	LAB treatment lowered the incidence of metritis and total uterine infections.	Deng et al., 2015
Performance	*Lactobacillus acidophilus Propionibacterium freudenreichii*	Dairy	Holstein cows	112	Treatments: (1) control; (2) 1 g/cow per day of 1 × 10^9^ cfu/g *Lactobacillus acidophilus* NP51 and 2 × 10^9^ cfu/g *Propionibacterium freudenreichii* NP24	10 weeks	Supplementing cows with DFM products did not affect cow performance	Ferraretto and Shaver, 2015
Performance	*Propionibacterium acidipropionici*	Beef	Heifers	20	Treatments: (1) Control; (2) *Propionibacterium acidipropionici* strain P169; (3) *P. acidipropionici* strain P5; (4) *Propionibacterium jensenii* strain P54. Inoculae of each strain (5 × 10^9^ cfu) were administered daily.	28 days	Total and major volatile fatty acid profiles were similar among all treatments. No effects were observed on dry matter intake and total tract digestibility of nutrients. Total enteric CH_4_ production (g/day) was not affected.	Vyas et al., 2014
Health	Propionibacterium *Lactobacillus plantarum Lactobacillus rhamnosus*	Sheep	Texel wethers	12	Treatments: (1) control; (2) *Propionibacterium* P63; (3) *L. plantarum* strain 115 plus P63 4) *L. rhamnosus* strain 32 plus P63. Treatment administered at a dose of 1 × 10^11^ cfu/wether/d.	24 days	LAB treatments may be effective in stabilizing ruminal pH and therefore preventing SARA risk, but they were not effective against lactic acidosis.	Lettat et al., 2012
Performance	*Lactobacillus acidophilus Propionibacterium freudenreichii*	Dairy	Holstein	60	Treatments: (1) control; (2) 4 × 10^9^ cfu/head *Lactobacillus acidophilus* NP51 and *Propionibacterium freudenreichii* NP24 (3) DFM plus glycerol	10 weeks	LAB treatments improved milk and protein yield, energy corrected milk	Boyd et al., 2011
Health and performance	*Lactobacillus plantarum*	Dairy	Female goats of Damascus breed	24	Goats were assigned to one of 2 treatments (1) 10^12^ cfu/day of *L. plantarum* PCA 236 (2) control	5 weeks	LAB treatment resulted in a decrease in fecal clostridia populations and a significantly higher content of polyunsaturated fatty acids in milk fat composition	Maragkoudakis et al., 2010
Health	*Lactococcus lactis*	Dairy	Holstein Friesian cows	6	5-ml suspension (containing 10^8^ cfu *L. lactis* DPC 3147) was infused into cow teat	400 h	Infusion with a live culture of a *L. lactis* lead to a rapid and considerable innate immune response.	Beecher et al., 2009
Performance	Propionibacterium	Dairy	Holstein cows	50	Treatments: (1) control; (2) Propionibacterium P169 at 6 × 10^11^ cfu per 25g of material	17 weeks	DFM supplementation did not increase milk production nor change milk composition but did increase feed efficiency	Weiss et al., 2008
Health	*Lactococcus lactis*	Dairy	Holstein-Friesian and New Zealand Friesians, Norwegian Reds, Normandes and Montbelliards.	Trial 1: 11; Trial 2:25	The injected suspension contained approximately 9. 1 ± 0. 5 ^10^ cfu/ml of *L. lactis* DPC3147	Trial 1: 2 weeks; Trial 2: 8 months	Of the 25 cases treated with the culture, 15 did not exhibit clinical signs of the disease following treatment. The results of these trials suggest that live culture treatment with *L. lactis* DPC3147 may be as efficacious as common antibiotic treatments in some instances.	Klostermann et al., 2008
Performance	*Lactobacillus acidophilus Propionibacteria freudenreichii*	Dairy	Holstein cows	57	Cows were randomly assigned to one of three diets. (1) 1 × 10^9^ cfu/d *L. acidophilus* strain LA747 and 2 × 10^2^ cfu/day *P. freudenreichii* strain PF2f. (2) 1 × 10^9^ cfu/day *L. acidophilus* strain LA747, 2 × 10^9^ cfu/day *P. freudenreichii* strain PF2f. (3) lactose (control)	28 days	Supplementing cows with DFM products did not affect cow performance, digestibility or rumen fermentation.	Raeth-Knight et al., 2007
Performance	*Propionibacterium*	Dairy	Holstein	44	Cows were randomly assigned to one of 3 treatments (1) control (2) 6 × 10^10^ cfu/cow of *Propionibacterium* P169 (3) 6 × 1011 cfu/cow of P169	30 weeks	DFM supplementation enhanced ruminal digestion of forage and early lactation cows receiving supplementation produced more milk but experienced a lower, but not depressed, fat percentage.	Stein et al., 2006
Performance	*Lactobacillus acidophilus Propionibacterium freudenreichii*	Beef	Steer cattle	Trial 1: 240 Trial 2: 660	Trial 1: four treatments (1) control, (2) 1 × 10^9^ cfu of *L. acidophilus* NP51 plus 1 × 10^6^ cfu of *L. acidophilus* NP45 plus 1 × 10^9^ cfu of *P. freudenreichii* NP24 per animal daily, (3) 1 × 109 cfu of *L. acidophilus* NP51 plus 1 × 10^9^ cfu of *P. freudenreichii* NP24 per animal daily (4) 1 × 10^6^ cfu of *L. acidophilus* NP51 plus 1 × 10^6^ cfu *L. acidophilus* NP45 plus 1 × 10^9^ cfu of *P. freudenreichii* NP24 per animal daily. Trial 2: three treatments (1) control (2) 5 × 10^6^ cfu of *L. acidophilus* NP51 plus 5 × 10^6^ cfu of *L. acidophilus* strain NP45 plus 1 × 10^9^ cfu of *P. freudenreichii* NP24 per animal daily (3) 1 × 10^9^ cfu of *L. acidophilus* NP51 plus 5 × 10^6^ cfu *L. acidophilus* NP45 plus 1 × 10^9^ cfu of *P. freudenreichii* NP24 per animal daily.	140 days	Overall, DFM supplementation did not greatly affect feedlot performance and carcass characteristics	Elam et al., 2003
Health	Propionibacterium *Enterococcus faecium*	Beef	Steer cattle	6	Treatments: (1) control, (2) *Propionibacterium* P15,(3) *Propionibacterium* P15 plus *Enterococcus faecium* EF212. Dose of 1 × 10^9^ cfu/g	20 days	DFM supplementation did not affect blood pH and blood glucose, however, steers fed the treatment had lower concentrations of blood CO_2_ than control steers, which is consistent with a reduced risk of metabolic acidosis.	Ghorbani et al., 2002
Performance	*Lactobacillus acidophilus Propionibacterium freudenreichii*	Beef	Heifers	450	Treatments: (1) control; (2) 5 × 10^8^ cfu/head/d *L. acidophilus* BG2FO4; (3) 1 × 10^9^ cfu/head/d *P. freudenreichii* P-63; (4) 5 × 10^8^ cfu/head/d *L. acidophilus* BG2FO4 and 1 × 10^9^ cfu/head/d *freudenreichii* P-63; (5) 5 × 10^8^ cfu/head/d *L. acidophilus* BG2FO4 and 1 × 10^9^ cfu/head/d P. *freudenreichii* P-63	126 days	Combined DFM supplementation resulted in significant improvements in daily gain and feed efficiency	Huck et al., 2000

*Trials related to the use of LAB supplementation to reduce shedding of *E. coli* O157:H7 in beef cattle are not listed but can be found in the meta-analysis performed by Wisener et al. (2015).*

LAB are the dominant silage inoculant in many parts of the world. LAB are used not only for their convenience and safety, but also because they are effective in controlling microbial events during silage fermentation (Muck et al., 2018). In the ensiling process, a succession of LAB ferment the available soluble sugars in cut plant material to produce organic acids, including lactic acid. As a result, the pH drops, preventing further microbial degradation of the plant material and preserving it as silage. The efficacy of adding LAB inoculants in enhancing the natural silage preservation process is well established. In addition, silage inoculants containing homofermentative LAB have not only improved silage quality and reduced fermentation losses but have also improved animal performance by increasing milk yield, daily gain and feed efficiency (Kung et al., 1993; Weinberg and Muck, 1996, 2013; Kung and Muck, 1997; Muck et al., 2018). The mechanism(s) behind the additional benefits in animal performance from feeding inoculated silage are not understood.

LAB DFMs and silage inoculants are microbial based technologies which are widely accepted and actively used in modern farming systems today. If LAB can be found to reduce ruminant CH_4_ production effectively then both DFMs and inoculants provide a practical and useful mitigation option on-farm.

### Methanogens and the Rumen

Rumen methanogenic archaea are much less diverse than rumen bacteria (Henderson et al., 2015), and members of two clades of the genus *Methanobrevibacter* (referred to as *M. gottschalkii* and *M. ruminantium*) make up ∼75% of the archaeal community (Janssen and Kirs, 2008; Henderson et al., 2015). Cultivated members of both of these methanogen clades are hydrogenotrophic and use H_2_ and CO_2_ for CH_4_ formation. Their cell walls contain pseudomurein and have similarities to those found in Gram positive bacteria which may be relevant to their sensitivity to antimicrobial agents (Varnava et al., 2017). Other significant members of the methanogen community in the rumen are methylotrophs, producing CH_4_ from methyl-containing substrates, particularly methylamines and methanol. These include strains of the genus *Methanosphaera* and members of the family *Methanomassiliicoccaceae*. The former have pseudomurein-containing cell walls, while the cell envelope surrounding the *Methanomassiliicoccaceae* has not been characterized. The ability of rumen bacteria to produce the H_2_ or methyl-containing substrates required for methanogenesis has been determined from culture studies, or is able to be inferred from genome sequences, but it is not yet known which bacteria are the most important contributors in the rumen.

**FIGURE 1:**
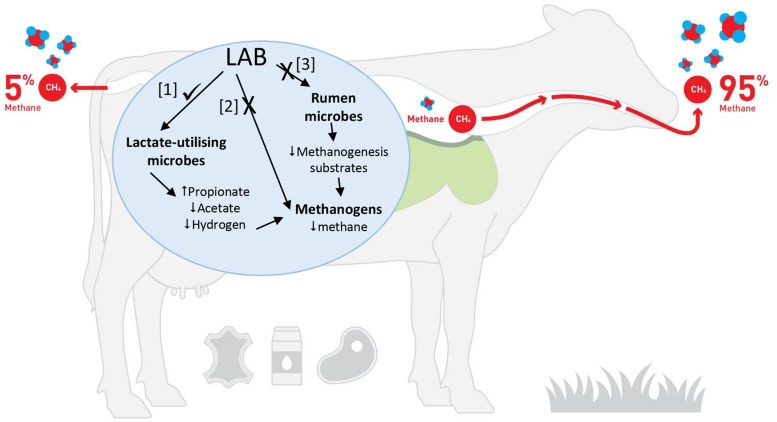
Potential pathways that could be modulated by LAB to decrease CH_4_ production [Adapted from FAO (2019). Image is being used with the permission of the copyright holder, New Zealand Agricultural Greenhouse Gas Research Centre (www.nzagrc.org.nz)].

How could LAB reduce ruminant CH_4_ production? It is hypothesized that LAB could influence ruminal methanogenesis in three possible ways (Figure 1): (1) use of LAB or their metabolites to shift the rumen fermentation so that there is a corresponding decrease in CH_4_ production, (2) use of LAB or their metabolites to directly inhibit rumen methanogens and (3) use of LAB or their metabolites to inhibit specific rumen bacteria that produce H_2_ or methyl-containing compounds that are the substrates for methanogenesis.

### How Have LAB Been Shown to Affect Ruminant CH_4_ Production?

The idea that LAB can be used to reduce CH_4_ production in ruminant livestock is not new. Reviews of CH_4_ mitigation strategies consistently refer to this possibility (Hristov et al., 2013; Takahashi, 2013; Jeyanathan et al., 2014; Knapp et al., 2014; Varnava et al., 2017). However, research on the topic has been limited and convincing data from animal trials to support this concept are lacking. Jeyanathan et al. (2016) screened 45 bacteria, including strains of LAB, bifidobacteria and propionibacteria, in 24h rumen *in vitro* batch incubations for their ability to reduce methanogenesis. Three strains were selected for *in vivo* trials in sheep (*n* = 12), and one strain (*Lactobacillus pentosus* D31) showed a 13% reduction in CH_4_ production (g CH_4_/kg/DMI) over 4 weeks when dosed at 6 × 10^10^ cfu/animal/day. The mechanism of action was not determined in this study, but the ability of introduced bacterial strains to persist in the rumen environment was highlighted as an important factor. Subsequent work by Jeyanathan et al. (2019) using the same strains has shown no ability to reduce CH_4_ emissions in dairy cows. A further two studies which examined LAB supplementation on CH_4_ production have had mixed results. Mwenya et al. (2004) assessed the effect of feeding *Leuconostoc mesenteroides* subsp. *mesenteroides* to sheep (*n* = 4). Supplementation with this strain was found to increase CH_4_ production (g CH_4_/kg/DMI) *in vivo*. The authors did not offer any discussion as to how a LAB strain could increase CH_4_ production *in vivo*. Astuti et al. (2018) evaluated 14 strains of *L. plantarum* in rumen *in vitro* experiments and identified strain U32 which had the lowest CH_4_ production value when compared to the other LAB treatment groups. The authors hypothesized the addition of LAB may have stimulated the growth of lactic utilizing bacteria leading to increased production of propionic acid and a subsequent decrease in the hydrogen availability for methane production (Astuti et al., 2018).

Research conducted on bacteriocins and their ability to reduce ruminal CH_4_ production has been minimal. The few bacteriocins and preparations from bacteriocin-producing lactic acid bacteria that have been examined have displayed promising results both *in vitro* and *in vivo*. Callaway et al. (1997) tested the effect of the *Lactococcus lactis* bacteriocin nisin on rumen fermentation *in vitro* and reported a 36% reduction in CH_4_ production. However, later work has shown nisin to be susceptible to rumen proteases limiting its potential efficacy *in vivo* (Russell and Mantovani, 2002). One *in vivo* trial has, however, reported a 10% decrease in CH_4_ emissions (g/kg DMI) in sheep (*n* = 4) fed this bacteriocin (Santoso et al., 2004). The trial was conducted for 15 days and the authors surmised that the reduction in CH_4_ was due to the inhibition of growth of the methanogenic microbes. Nollet et al. (1998) examined the addition of the cell-free supernatant of *Lactobacillus plantarum* 80 (LP80) to ruminal samples *in vitro* and noted an 18% decrease in CH_4_ production and a 30.6% reduction in CH_4_ when the supernatant was combined with an acetogenic culture, *Peptostreptococcus productus* ATCC 35244. The effect of the LP80 supernatant in combination with *P. productus* was also studied *in vivo* using two rams and it was concluded that inhibition of methanogenesis (80% decrease; mmol/6 h) occurred during the first 3 days but the effect did not persist. Compounds (PRA1) produced by *L. plantarum* TUA1490L were tested *in vitro* and found to decrease methanogenesis by 90% (Asa et al., 2010). Further work with PRA1 confirmed its ability to maintain an antimicrobial effect even after incubation with proteases but the hypothesis that the inhibition mechanism of PRA1 may relate to the production of hydrogen peroxide has not been proven (Takahashi, 2013). Bovicin HC5, a bacteriocin produced by *Streptococcus equinus* HC5, inhibited CH_4_ production by 53% *in vitro* (Lee et al., 2002), while more recently the bacteriocin pediocin produced by *Pediococcus pentosaceus* 34 was shown to reduce CH_4_ production *in vitro* by 49% (Renuka et al., 2013). The possibility of using bacteriocins from rumen streptococci for CH_4_ mitigation has recently been reviewed (Garsa et al., 2019). Currently, it is not clear whether the bacteriocins affect the methanogens themselves, or whether they affect the other rumen microbes that produce substrates necessary for methanogenesis. The only evidence that bacteriocins affect methanogens directly is a single article (Hammes et al., 1979) in which nisin was shown to inhibit a non-rumen methanogen, *Methanobacterium*, using an agar diffusion assay to determine the inhibitory effect. Recently, Shen et al. (2017) used *in vitro* assays and 16S rRNA gene analysis to assess the effect nisin has on rumen microbial communities and fermentation characteristics. Results demonstrate that nisin treatments can reduce populations of total bacteria, fungi and methanogens resulting in a decrease in the ratio of acetate to propionate concentrations. A similar class of compounds (antimicrobial peptides such as human catelicidin) have also been shown to be strongly inhibitory to a range of methanogens (Bang et al., 2012, 2017). There is no standardized approach to screening methanogen cultures for their susceptibility to bacteriocins, however, the method developed to facilitate screening of small molecule inhibitors (Weimar et al., 2017) should be useful. This employs the rumen methanogen strain AbM4 (a strain of *Methanobrevibacter boviskoreani*) which grows without H_2_ in the presence of ethanol and methanol (Leahy et al., 2013).

Many LAB silage inoculants possess antibacterial and/or antifungal activity and in some cases this activity is imparted into the inoculated silage (Gollop et al., 2005). The inhibitory activity has been shown to inhibit detrimental micro-organisms in silage (Flythe and Russell, 2004; Marciňáková et al., 2008; Amado et al., 2012) and has been postulated to do the same in the rumen, but the role of specific silage inoculants in CH_4_ mitigation has received little attention. Thus far, research has demonstrated that LAB included in freeze-dried silage inoculants can survive in rumen fluid (Weinberg et al., 2003) and that LAB survive passage from silage into rumen fluid *in vitro* (Weinberg et al., 2004). Several studies have demonstrated that *in vitro* rumen fermentation can be altered by some LAB strains. Muck et al. (2007) made silages using a range of inoculants and showed *in vitro* that some of the inoculated silages had reduced gas production compared with the untreated silage suggesting a shift in fermentation had occurred. Cao et al. (2010a) investigated the effect of *L. plantarum* Chikuso-1 on an ensiled total mixed ration (TMR) and showed CH_4_ production decreased by 8.6% and propionic acid increased by 4.8% compared with untreated TMR silage. Cao et al. (2011) found similar results with the same inoculant strain in vegetable residue silage with the inoculated silage having higher *in vitro* dry matter digestibility and lower CH_4_ production (46.6% reduction). Further work with this LAB strain *in vivo* showed that the inoculated TMR silage increased digestibility and decreased ruminal CH_4_ (kg DMI) emissions (24.7%) in sheep (*n* = 4) compared with a non-inoculated control (Cao et al., 2010b). Although more research is required in this area, the results suggest that some LAB strains are capable of altering ruminal fermentation leading to downstream effects such as reduced CH_4_ production.

## Conclusion and Future Perspectives

Literature on the use of LAB to reduce CH_4_ production in ruminants is limited. In the small number of studies available, *in vitro*, LAB can reduce CH_4_ production effectively. The effect is clearly strain dependent and it is not understood whether the LAB or their metabolites affect the methanogens themselves, or whether they affect the other rumen microbes that produce substrates necessary for methanogenesis. *In vivo*, the lack of robust animal trials (appropriate animal numbers, relevant treatment groups, trial period, and strain efficacy) investigating LAB supplementation and CH_4_ mitigation make it impossible at this time to make a comprehensive conclusion. Much more research is needed to understand the mechanisms behind the use of LAB as rumen modifiers. However, if appropriate LAB cultures can be identified, and proven to be effective *in vivo* then a range of delivery options that are already accepted in the global farming system such as DFMs and silage inoculants are available. This represents an alternative approach to CH_4_ mitigation research and one that can be used in combination with other mitigation options such as vaccines (Wedlock et al., 2013) and CH_4_ inhibitors (Dijkstra et al., 2018) which are currently under development. Ruminant production systems with low productivity lose more energy per unit of animal product than those with high productivity. In systems where farm management practices result in an increase in performance per animal (e.g., kg milk solids per cow, kg lamb slaughtered per ewe, kg beef slaughtered per cow), and combined with a reduction in stocking rates, then absolute CH_4_ emissions can be reduced. LAB supplementation and use of silage inoculants can contribute to these on-farm management options that reduce agricultural GHG emissions through increases in animal productivity and improved health. LAB supplementation could offer a practical, effective and natural approach to reducing CH_4_ emissions from ruminant livestock and contribute to the on-farm management practices that can be used to reduce CH_4_ emissions.

## Author Contributions

SL, WK, and GA conceived the research. ND, PM, WK, YL, and SL performed the analysis and wrote the manuscript. RR, CS, and GA reviewed the final manuscript.

## Conflict of Interest

WK was employed by the company Donvis Ltd. The authors declare that the research was conducted in the absence of any commercial or financial relationships that could be construed as a potential conflict of interest.
